# Targeting tumor heterogeneity: multiplex-detection-based multiple instance learning for whole slide image classification

**DOI:** 10.1093/bioinformatics/btad114

**Published:** 2023-03-02

**Authors:** Zhikang Wang, Yue Bi, Tong Pan, Xiaoyu Wang, Chris Bain, Richard Bassed, Seiya Imoto, Jianhua Yao, Roger J Daly, Jiangning Song

**Affiliations:** Monash Biomedicine Discovery Institute, Department of Biochemistry and Molecular Biology, Monash University, Melbourne, VIC 3800, Australia; Monash Biomedicine Discovery Institute, Department of Biochemistry and Molecular Biology, Monash University, Melbourne, VIC 3800, Australia; Monash Biomedicine Discovery Institute, Department of Biochemistry and Molecular Biology, Monash University, Melbourne, VIC 3800, Australia; Monash Biomedicine Discovery Institute, Department of Biochemistry and Molecular Biology, Monash University, Melbourne, VIC 3800, Australia; Department of Human Centred Computing, Faculty of Information Technology, Monash University, Melbourne, VIC 3800, Australia; Victorian Institute of Forensic Medicine, Melbourne, VIC 3006, Australia; Division of Health Medical Intelligence, Human Genome Center, The Institute of Medical Science, The University of Tokyo, Tokyo 108-8639, Japan; Tencent AI Lab, Tencent, Shenzhen, Guangdong 518000, China; Monash Biomedicine Discovery Institute, Department of Biochemistry and Molecular Biology, Monash University, Melbourne, VIC 3800, Australia; Monash Biomedicine Discovery Institute, Department of Biochemistry and Molecular Biology, Monash University, Melbourne, VIC 3800, Australia

## Abstract

**Motivation:**

Multiple instance learning (MIL) is a powerful technique to classify whole slide images (WSIs) for diagnostic pathology. The key challenge of MIL on WSI classification is to discover the *critical instances* that trigger the bag label. However, tumor heterogeneity significantly hinders the algorithm’s performance.

**Results:**

Here, we propose a novel multiplex-detection-based multiple instance learning (MDMIL) which targets tumor heterogeneity by multiplex detection strategy and feature constraints among samples. Specifically, the internal query generated after the probability distribution analysis and the variational query optimized throughout the training process are utilized to detect potential instances in the form of internal and external assistance, respectively. The multiplex detection strategy significantly improves the instance-mining capacity of the deep neural network. Meanwhile, a memory-based contrastive loss is proposed to reach consistency on various phenotypes in the feature space. The novel network and loss function jointly achieve high robustness towards tumor heterogeneity. We conduct experiments on three computational pathology datasets, e.g. CAMELYON16, TCGA-NSCLC, and TCGA-RCC. Benchmarking experiments on the three datasets illustrate that our proposed MDMIL approach achieves superior performance over several existing state-of-the-art methods.

**Availability and implementation:**

MDMIL is available for academic purposes at https://github.com/ZacharyWang-007/MDMIL.

## 1 Introduction

Whole slide imaging, which refers to scanning and converting a complete microscope slide to a digital whole slide image (WSI), is an efficient technique for visualizing tissue sections in disease diagnosis, medical education, and pathological research (Pantanowitz et al. 2011; Cornish et al. 2012). In recent years, with the advances in artificial intelligence and big data techniques, WSI-based computational pathology has attracted increasing attention due to its clinical and commercial value in precision medicine. Remarkable successes have been achieved in combing medical image analysis, machine learning, and deep learning (Yu et al. 2016; Campanella et al. 2019; Bulten et al. 2020; Lu et al. 2021; Wang et al. 2021a, 2022a; Zeng et al. 2021).

Typically, the gigapixel WSIs with a size of about 40 000 × 40 000 pixels are computationally infeasible with current hardware. Pinckaers et al. (2021) train an end-to-end deep neural network which takes the entire WSIs as input directly by utilizing a streaming implementation of convolutional layers. Even with the specific designs, this method still suffers from relatively low accuracy and high computational requirements. Accordingly, patch-based processing approaches (Cruz-Roa et al. 2014; Mousavi et al. 2015; Hou et al. 2016; Maksoud et al. 2020), which divide each WSI into thousands of small patches/tiles and utilize the neural networks for examination, have become a mainstream practice. Considering the patch-level labeling of WSIs by pathologists is time-consuming and challenging, weakly supervised multiple instance learning (MIL), which eliminates the laborious process of instance labeling by assuming an entire bag with one label, dominates in this area (Ilse et al. 2018; Campanella et al. 2019; Li et al. 2021; Shao et al. 2021).

Currently, most MIL methods are modeled with self/cross-attention mechanisms, Transformers, or Graph Neural Networks. For example, Ilse et al. (2018) and Li et al. (2021) propose DeepMIL and DSMIL, which introduce the attention and cross-attention mechanisms into the algorithms, respectively. DeepMIL directly computes an attention score for each instance and conducts weighted aggregation. However, this strategy typically cannot detect critical instances and has low generalization ability. DSMIL selects one representative instance as a query to detect the potential ones through a cross-attention operation. However, their instance selection strategy is unreliable and significantly limits the upper bound of the algorithm performance. Furthermore, due to tumor heterogeneity, one instance from the WSI is often not representative enough. Shao et al. (2021) propose TransMIL, which introduces the Transformer architecture into the model. They utilize a single classification token together with the multi-head self-attention module (MHSA) to extract valuable features from the whole bag. Compared with attention-based methods, TransMIL is theoretically more reasonable. The limitation of TransMIL lies in the computational burden of building contextual connections. Besides, all these methods do not consider the challenges of tumor heterogeneity and often do not generalize well on the testing data. Zhang et al. (2022) enrich the data by double-tier augmentation and achieve good performance by distillation technology. To some degree, they increase the training data diversity but still fail to address the tumor heterogeneity challenge from the modeling perspective. Therefore, algorithms specifically designed for tumor heterogeneity are urgent.

To tackle the above issue, we propose a novel approach termed multiplex-detection-based multiple instance learning (MDMIL). MDMIL is developed based on the internal query (IQ) generation module (IQGM) and multiplex detection module (MDM) and assisted by the memory-based contrastive loss during the training phase. Specifically, IQGM generates the probability distribution of instances on deep-transferred instance features through a classification layer and generates the IQ by aggregating highly reliable instances after the probability analysis. Then, MDM, which consists of the multiplex-detection cross-attention (MDCA, cross-attention module) and multi-head self-attention (Vaswani et al. 2017) (MHSA, self-attention module), is used to generate the final representations for WSIs. In this process, the IQ and predefined learnable variational query (VQ) jointly support the instance detection in the form of internal and external assistance. Both IQ and VQ have their own characteristics and can be complementary on functions to some degree, thus the multiplex detection strategy (jointly utilize IQ and VQ for detection) significantly improves the instance mining and generalization capacities of the network. Then, MHSA establishes the communications of the representations corresponding to different subtypes, reducing overlapping in the feature space and highlighting the critical characteristics. In addition, inspired by the recent success of the self-supervised learning approach MoCo (He et al. 2020), we adopt the memory-based contrastive loss into the training phase. With this loss function, we can achieve consistency on various phenotypes in the feature space, thereby relieving the challenges caused by tumor heterogeneity and increasing the training stability. To the best of our knowledge, this represents the first study to leverage memory-based metric learning to improve the WSI classification performance. We evaluate our proposed MDMIL for WSI classification on three benchmarks, e.g. CAMELYON16, The Cancer Genome Atlas non-small cell lung cancer (TCGA-NSCLC), and TCGA renal cell carcinoma (TCGA-RCC) datasets. The experimental results demonstrate its state-of-the-art performance over other methods by a large margin. Meanwhile, the ablation study verifies that each proposed module can work independently and cooperatively.

## 2 Related work

### 2.1 Application of MIL in WSI classification

There are basically two categories of MIL methods in WSI classification: instance-level and embedding-level algorithms. The instance-level algorithms (Campanella et al. 2019; Xu et al. 2019) assign the WSI label to the patches during model training. Then, the top instances are selected for aggregation and prediction. This strategy can introduce a large number of noisy features and result in bad model convergence and performance. As for the embedding-level algorithms, each patch is mapped to a feature vector; then, the feature vectors are aggregated using various techniques. A number of attention-based MIL algorithms (Ilse et al. 2018; Lu et al. 2021) have been proposed owing to the prevalence of attention mechanisms. Specifically, such methods calculate an attention score for each instance with trainable parameters and then conduct a weighted sum. These methods eliminate patch-level labels and enable prediction through global characteristics. Besides, feature clustering methods (Xie et al. 2020; Sharma et al. 2021) generate the final representations by calculating and aggregating the cluster centroids of all the feature embeddings. Recently, transformer architecture (Vaswani et al. 2017), which has the context-aware ability, has also been adopted in MIL to learn the instance correlations. Shao et al. (2021) propose a transformer-based WSI classification model, which comprehensively considers the correlations among different instances within the same bag. However, that model relies on the trainable parameters with less prior knowledge from the WSIs, thereby failing to achieve remarkable performance progress.

### 2.2 Attention in deep learning

In neural networks, the attention mechanism is a technique that mimics human cognition. Initially, the attention mechanism was used to extract meaningful information from sentences in machine translation (Bahdanau et al. 2014); the attention weights intuitively show how the network adjusts its focus according to the context. In recognition of its success in natural language processing, many researchers employ it to tackle natural image tasks, e.g. image classification (Hu et al. 2018), and person reidentification (Wang et al. 2021b). Essentially, these methods give appropriate weights to the spatial, channel, or temporal dimensions to emphasize valuable features and discard noise. Recently, many MIL algorithms have adopted the attention mechanism for feature aggregation and achieved good performance.

Transformer prevails in many different areas (Vaswani et al. 2017; Yuan et al. 2021; Wang et al. 2022b) due to its outstanding ability and unique attention mechanism. The original transformer (Vaswani et al. 2017) in NLP is a novel architecture aiming to solve sequence-to-sequence tasks while handling long-range dependencies. It relies entirely on the self-attention mechanism to compute its output representations. The vision transformer (ViT) (Yuan et al. 2021) emerged as a competitive alternative to convolutional neural networks (CNNs) that are currently state-of-the-art in computer vision and accordingly have been widely used in different image recognition tasks. In this article, our proposed MDMIL framework is also derived from the transformer architecture.

## 3 Methodology

**Figure 1:**
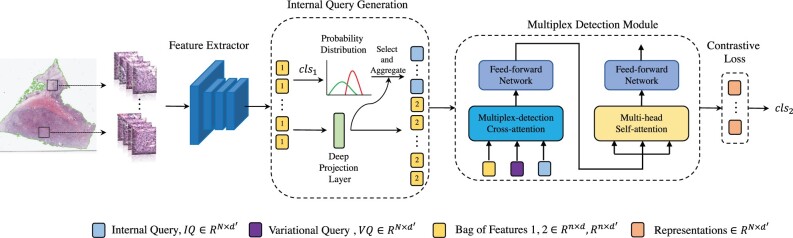
Overview of the proposed multiplex-detection-based multiple instance learning. We first adopt a segmentation algorithm to discard the background and crop the tissue regions into patches. Secondly, the patches are embedded into feature vectors with a pre-trained feature extractor. Then, the bag of features is fed into the internal query generation module (IQGM), deep projection layer (DPL), and multiplex detection module (MDM) to generate the final representation of the WSI. Finally, the classification layer generates the final prediction result. Here, the yellow blocks 1 and 2 refer to the bag of features preceding and following the deep projection layer, respectively

This section briefly introduces the proposed multiplex-detection-based multiple instance learning (MDMIL). The overall architecture is illustrated in Fig. 1. Firstly, we segment the tissue region of each slide through an automated segmentation algorithm and divide it into patches with a fixed size. Secondly, we adopt the ImageNet pre-trained model to extract features of patches for a fair comparison with previous works. Following Lu et al. (2021) and Shao et al. (2021), we only adopt the first *Convolution Block* and the first three *Residual Blocks* of the ResNet50 (He et al. 2016) as the extractor; therefore, each patch is encoded as a 1024D feature vector, while the bag of instances (each WSI) can be represented as $F\in R^{n\times 1024}$, where *n* varies depending on the number of patches in each WSI. After converting all tissue patches into low-dimensional feature embeddings, training and inference can occur in the low-dimensional feature space instead of the high-dimensional pixel space. Thirdly, the proposed IQGM analyzes the probability distribution of instances and generates the IQ for MDM. Next, the MDM detects the critical instances and aggregates the final representations for the WSI. Meanwhile, we utilize the memory-based contrastive loss in the training phase to enforce distance constraints in the feature space. Details of each module will be presented in the following section.

### 3.1 IQ generation module

Our proposed IQGM aims to generate the IQ as the internal assistance for the subsequent MDM. In DSMIL (Li et al. 2021), they utilize a classification layer *cls* together with a *softmax* function and a max-pooling operation to retrieve features with the highest probability corresponding to each subtype. However, this strategy has two obvious shortcomings: (i) the prediction accuracy of *cls* is relatively low, and the absolute predictive power of the whole model will be logically limited by the upper bound of *cls’*s expression; (ii) due to the application of deep transfer learning and the tumor heterogeneity, the retrieved features tend to be short of class-level representative and have much patch-specific information.

A straightforward approach to tackle the above issues is averaging the top instance features of each subtype. However, this approach will introduce noise with a large chance, thus affecting the convergence of the model and the stability of the model training.

As shown in Fig. 1, IQGM has two parallel streams. In the lower stream, we employ a deep projection layer (DPL) constructed by one nonlinear projection unit and one linear projection layer to achieve feature recalibration. After that, we obtain $F^{\prime }\in R^{n\times d^{\prime }}$ and $f_{i}^{\prime }\in R^{1\times d^{\prime }}$. The nonlinear projection unit consists of one fully connected layer, one layer normalization (*LN*) (Ba et al. 2016), and one *ReLU* activation function. DPL is essential for the network because it can project the features of similar tissue sections to a relatively compact cluster in the feature space, thereby facilitating feature mining in the subsequent attention operations. In the upper stream, we adopt a classification layer $cls_{1}$ and a *softmax* function to calculate the probability distribution of instances as follows:


(1) $$P=\mathrm{softmax}(cls_{1}(\{f_{1},\ldots ,f_{n}\})).$$

Then, we define a confidence factor $cf_{i}$ for the subtype *i* through the mean value ${\bar{m}}_{i}$ and standard deviation $\sigma_{i}$ of the top $K_{1}$ instances:


(2) $$cf_{i}=\bar{m_{i}}-\sigma_{i};\bar{m_{i}}=\frac{1}{K_{1}}\sum_{j=1}^{K_{1}}p_{ij};\;\;\sigma_{i}=\sqrt{\frac{\sum_{j=1}^{K_{1}}{\left(p_{ij}-{\bar{m}}_{i}\right)}^{2}}{K_{1}}},.$$

Suppose that the maximum one $cf_{\text{max}}$ satisfies the following requirement: where β∈[−1,1] is a hyperparameter for alleviating the instance imbalance issue. In that case, we consider the subtype of $cf_{\text{max}}$ as a confident one and aggregate the top $K_{1}$ corresponding instance features of $F^{\prime }$ through a mean function as the reliable IQ $q_{rb}$. As for other subtypes, we only aggregate the top $K_{2}$ corresponding instance features as *q*. If no $cf_{\text{max}}$ exists, there will be no reliable query and we only average the top $K_{2}$ instance features. Here, $K_{1}$ and $K_{2}$ are not fixed numbers; they are the multiplication of the bag length and predefined ratio hyperparameter $r_{1},r_{2}\in [0,1]$. Typically, $r_{1}$ is bigger than $r_{2}$ as the former involves more instance information than the latter. We discuss parameters along with the datasets in Section 4. The pipeline of the algorithm is shown in Algorithm 1.


(3) $$cf_{\text{max}}-\beta >\forall \{cf_{1},\ldots ,cf_{N};\text{index}\neq \text{max}\},$$

Using the proposed IQGM, we can generate a reliable IQ with less noise, thus laying a good foundation for the following prediction.

Algorithm 1 Internal query generation module

**Input:**

A bag of feature embeddings $F=f_{1},\ldots ,f_{n}$, where $F\in R^{n\times d}$ and $f_{i}\in R^{1\times d}$.

**Output:**

Reliable IQ of each subtype;

1:  Get the transformed features with DPL;

$F^{\prime }=\text{DPL}(F)=\text{DPL}(f_{1},\ldots ,f_{n})$

2: Get the probability distribution of instances through a classification layer;

$P\leftarrow \mathrm{softmax}(cls_{1}(\{f_{1},\ldots ,f_{n}\}));$

3: Calculate the confidence factor for each subtype through the top $K_{1}$ instances;

$CF\leftarrow \{cf_{i}=\bar{m_{i}}-\sigma_{i};i=1,\ldots ,N\};$

4: Estimate the IQ;

if $cf_{\text{max}}-\beta >\forall \{cf_{1},\ldots ,cf_{N};\text{index}\neq \text{max}\}$:

  Average the top $K_{1}$ and $K_{2}$ instances for confident and other subtypes, respectively

$\text{IQ}=\{q_{rb},q_{i};\text{index}\neq \text{max}\}$

else:

  Average the top $K_{2}$ instances for all the subtypes

$\text{IQ}=\{q_{1},\ldots ,q_{N}\}$

### 3.2 Multiplex detection module

**Figure 2:**
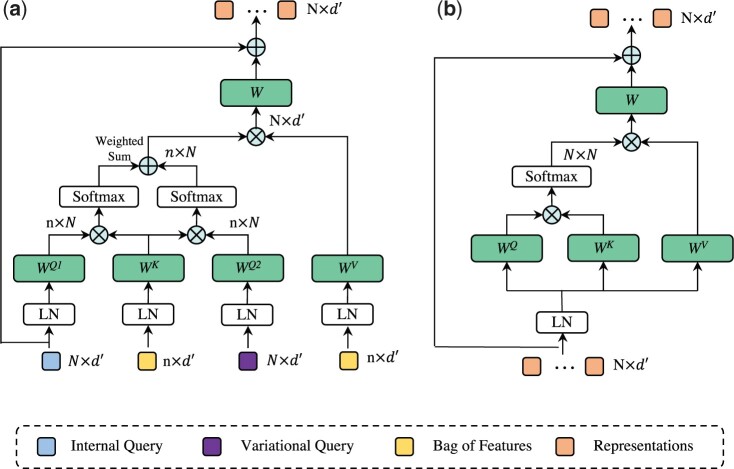
Overview of the proposed multiplex-detection cross attention (MDCA) (a) and traditional multi-head self-attention (MHSA) (b). The main differences between the two modules lay in the input and the attention strategy. There are two *Queries* in MDCA, and MHSA arises *Query*, *Key*, and *Value* from one input

The proposed MDM aims to detect the critical instances that trigger the prediction under tumor heterogeneity circumstances. Previous methods adopt either an unreliable IQ (Li et al. 2021) or a single VQ (classification token) (Shao et al. 2021) to detect and aggregate the features from bags, failing to create a robust algorithm due to their inferior feature mining ability. Here, we integrate the two strategies within one module, enabling the proposed module to achieve superior performance through internal and external assistance. As shown in Fig. 1, MDM comprises one multiplex-detection cross-attention (MDCA), one multi-head self-attention (MHSA), and two feed-forward networks (FFN) (Xiong et al. 2020). We illustrate the MDCA and MHSA in Fig. 2a and b, respectively.

Essentially, MDCA is a modified cross-attention module based on the standard architecture of the MHSA. MDCA has three inputs: IQ from IQGM, trainable VQ, and the bag of features $F^{\prime }$ after the DPL. Given the feature vector, *query one* $Q_{1}$ arises from the IQ, *query two* $Q_{2}$ arises from the VQ, and *key K* and *value V* arise from the $F^{\prime }$. Formally, where $W\in R^{d^{\prime }\times d^{\prime }}$ denotes linear projection. We retain the multi-head strategy in MHSA and split the features into *m* parts along the channel dimension, and get $Q^{1},Q^{2}\in R^{N\times m\times (d^{\prime }/m)}$ and $K,V\in R^{n\times m\times (d^{\prime }/m)}$. Next, we calculate two attention matrices ($mt^{1}$ and $mt^{2}$) based on $Q^{1}$ and $Q^{2}$, respectively. The function for each matrix can be presented as: where $\sqrt{d_{k}}$ is a scaling factor and, *i* and *j* represent the indices of *query* and *key*, respectively. Each element of the attention matrix indirectly indicates the similarity between *query* and *key*. We take the weighted sum of $mt_{1}$ and $mt_{2}$ as the final attention matrix $m^{\prime }$. We define the weights of the two matrices according to the reliability of IQ. If the critical instances cover a large area of the WSI and can be readily found, IQ will be assigned more attention for fast model convergence. Otherwise, more attention will be assigned to the VQ. The aggregation process can be defined as:


(4) $$\begin{array}{c} Q_{1}=LN(\text{IQ})W^{1},Q_{2}=LN(\text{VQ})W^{2},\\ K=LN(F^{\prime })W^{3},V=LN(F^{\prime })W^{4}, \end{array}$$

(5) $$mt_{i,j}=\frac{\;\text{exp}(\beta_{i,j})}{\sum_{j=1}^{n}\text{exp}(\beta_{i,j})},\;\beta_{i,j}=\frac{Q_{i}K_{j}}{\sqrt{d_{k}}},$$

(6) $$\begin{array}{c} f_{ag}=\mathrm{MDCA}(Q^{1},Q^{2},K,V)\\ =(\alpha \cdot mt_{1}+(1-\alpha )\cdot mt_{2})\times V. \end{array}$$

Then, we conduct a projection operation with a linear layer. At last, we perform the residual operation between the attention output and IQ to avoid the gradient vanishing. The final output of MDMC is $f_{ag}\in R^{N\times d^{\prime }}$, where *N* is the number of subtypes. With the cross-attention between IQ and *K*, we successfully establish the connections between all instances and a collection of potential critical ones. Through the cross-attention between VQ and *K*, we further supplement other features to improve the model’s robustness. Besides, since IQ is derived from the bag of features, it converges fast and can provide internal assistance for feature detection. As for VQ, it is optimized through the whole training set, providing external and global assistance for feature detection. Thus, the two kinds of *queries* can be complementary with each other; their combination can greatly improve the model’s performance. Moreover, with the cross attention, we can considerably reduce the instance-dimension from the original patch number *n* (typically around 10 000) to subtype number *N*, requiring much less computation in the following calculation than other transformer-based methods, e.g. TransMIL (Shao et al. 2021). After the cross-attention operation, we employ the post-layer normalization feed-forward network (FFN) (Xiong et al. 2020) to conduct a non-linear transformation. We describe the details of FFN in Supplementary Materials Section S1).

Next, we employ the MHSA (Vaswani et al. 2017) (as shown in Fig. 2b) to establish the communications of the representations corresponding to different subtypes. The self-attention module does not require any external information and can compute independently. Through the self-attention operation, we can further disentangle the representations and guarantee less overlap in the feature space. Finally, we utilize another nonlinear transformation FFN to recalibrate the representations. After generating the features that correspond to all subtypes, we simply average all the features to get the final representation of the WSI.

### 3.3 Memory-based contrastive loss

Metric learning is very effective for optimizing deep neural networks. However, since WSIs have different numbers of instances and only one bag of features can be fed into the neural network in each iteration, metric learning by referring to other samples within the mini-batch would be infeasible for WSI-based tasks. Recently, memory-based methods have been widely adopted in self-supervised learning (He et al. 2020) and other computer vision tasks (Wang et al. 2022b). Inspired by these methods, we adopt a memory-based contrastive loss to promote network performance, which can implement constraints in the feature space by referring to the whole training data.

There are two steps for the loss function: memory initialization at the beginning of the algorithm and memory update throughout the training process. The memory is initialized with the feature centers in the training set. Firstly, we perform the forward computation to generate the labeled subtype representation. Then, we conduct the average and l2 normalization operations for features of each subtype separately to obtain the feature centers $C=\{c_{1},\ldots ,c_{N}\}$. In terms of the memory update, the *k*_th_ center $c_{k}$ is updated during training by a momentum controlled as follows: where *m* is the momentum coefficient and $f_{i}$ is the labeled subtype representation. Functionally, the *Contrative Loss* is defined as: where τ is a predefined temperature parameter and $c_{i}$ represents the feature center with an identical subtype, respectively.


(7) $$c_{k}=mc_{k}+(1-m)f_{i},$$

(8) $$L_{\text{CL}}=-\text{log}\frac{\;\text{exp}(<f,c_{i}>/\tau )}{\sum_{j}^{N}\text{exp}(<f,c_{j}>/\tau )},$$

### 3.4 Overall loss

There are three loss functions during the training phase: two cross-entropy loss ($L_{\text{CEL}}$) and one contrastive loss ($L_{\text{CL}}$). We calculate $L_{\text{CEL}}$ with the classification output $P_{1}$ and $P_{2}$ from IQGM and the final classification layer. Notably, we adopt a max-pooling operation on $P_{1}$ to get the instance with the highest score for loss calculation. Functionally, $L_{\text{CEL}}$ can be presented as: where *W* is a linear projection matrix, $y_{i}$ is the corresponding label and *N* is the total number of classes.


(9) $$L_{\text{CEL}}=-y_{i}\;\text{log}(\frac{\;\text{exp}(W_{i}f_{i})}{\sum_{j=1}^{N}\text{exp}(W_{j}f_{j})}),$$

Therefore, the overall loss functions can be expressed as: where α is a predefined soft parameter and is 0.5.


(10) $$\begin{array}{c} L_{\text{Final}}=L_{\text{CEL}1}(\text{max-pooling}(P_{1}),y)\\ +L_{\text{CEL}2}(P_{2},y)+\alpha L_{\text{CL}}, \end{array}$$

## 4 Experiments and results

In this study, we demonstrate the superior performance of the proposed MDMIL on three public datasets: The Cancer Genome Atlas non-small cell lung cancer (TCGA-NSCLC), TCGA-RCC, and CAMELYON16.

### 4.1 Dataset

TCGA-NSCLC dataset has two subtypes: lung squamous cell carcinoma (TCGA-LUSC) and lung adenocarcinoma (TCGA-LUAD). There are 993 diagnostic WSIs, including 507 LUAD slides from 444 cases and 486 LUSC slides from 452 cases, respectively. After processing, the mean number of patches extracted per slide at ×20 magnification is 11 038.

The TCGA-RCC dataset has three subtypes: kidney chromophobe renal cell carcinoma, kidney renal clear cell carcinoma, and kidney renal papillary cell carcinoma. There are 884 diagnostic WSIs, including 111 KICH slides from 99 cases, 489 KIRC slides from 483 cases, and 284 KIRP slides from 264 cases. After processing, the mean number of patches extracted per slide at ×20 magnification is 12 386.

CAMELYON16 is a public dataset of metastasis in breast cancer. There are 270 WSIs in the training set, including 159 normal tissues and 111 tumor tissues. As for the testing set, there are in total of 130 WSIs. After processing, the mean number of patches extracted per slide at ×20 magnification is 9752.

### 4.2 Experiment setup

Following Shao et al. (2021), we crop each WSI into a series of 256 × 256 non-overlapping patches and discard the background patches (saturation < 15). For the TCGA datasets, we randomly split the whole dataset into non-overlapping training, validation, and testing datasets at the slide level with a ratio of 60%, 15%, and 25%. While for the CAMELYON16 dataset, it has an official testing set; we respectively take 90% and 10% WSIs of the training set for training and validation. All the following experiments adopt this split protocol. The best-performing model on the validation set will be taken for testing. In addition, for a fair comparison, the input instance features for all experiments are extracted by modified ResNet50 (one *Convolutional Block* and three *Residual Blocks*).

We provide a detailed description of the implementation details and evaluation metrics in Supplementary Material Section S2).

### 4.3 Results on WSI classification

We evaluate our proposed model on both detection and subtype classification datasets. For the detection dataset, e.g. CAMELYON16, the positive WSI contains metastases while the negative ones do not contain metastases. As for the subtype classification datasets, e.g. TCGA-NSCLC and TCGA-RCC, each subtype has its unique pattern. We present all the experimental results in Table 1.

**Table 1:** Performance comparison with other state-of-the-art methods on CAMELYON16, TCGA-NSCLC, and TCGA-RCC datasets in terms of accuracy and AUC. The best results of each dataset are marked in bold.

	CAMELYON16		TCGA-NSCLC		TCGA-RCC	
	Accuracy	AUC	Accuracy	AUC	Accuracy	AUC
ABMIL (Ilse et al. 2018)	0.8682	0.8760	0.7719	0.8656	0.8934	0.9702
PT-MTA (Li et al. 2019)	0.8217	0.8454	0.7379	0.8299	0.9059	0.9700
DSMIL (Li et al. 2021)	0.7985	0.8179	0.8058	0.8925	0.9294	0.9841
CLAM-SB (Lu et al. 2021)	0.8760	0.8809	0.8180	0.8818	0.8816	0.9723
CLAM-MB (Lu et al. 2021)	0.8372	0.8679	0.8422	0.9377	0.8966	0.9799
TransMIL (Shao et al. 2021)	0.8837	0.9309	0.8835	**0.9603**	0.9466	0.9882
DTFD-MIL (AFS) (Zhang et al. 2022)	0.8954	0.9410	0.8895	0.9487	0.9376	0.9717
Ours	**0.9158**	**0.9669**	**0.9052**	0.9596	**0.9693**	**0.9944**

**CAMELYON16**: Since the positive slides only contain a small portion of metastasis tissue (roughly < 10% of the tissue area), thereby CAMELYON16 is one of the most challenging datasets to evaluate the effectiveness of the MIL algorithm. Only algorithms with good generalization capability can perform well on this dataset. Considering the instance distribution of the dataset, we set $r_{1}$ and $r_{2}$ as 0.05 and 0.01, respectively. As shown in Table 1, our method achieves an Accuracy of 0.9158 and AUC of 0.9669, respectively. Especially in terms of AUC, our method outperforms TransMIL (Shao et al. 2021) and DTFD-MIL (Zhang et al. 2022) by 3.6% and 2.0%, respectively. TransMIL (Shao et al. 2021) utilizes the MHSA to bridge the connections between instances and adopt the classification token to aggregate the critical features. As it is difficult for the classification token to collect all the valuable features, the algorithm lacks generalization on the testing set. DSMIL (Li et al. 2021) is based on *i.i.d* hypothesis. They utilize one retrieved IQ to aggregate the bag of features, which results in bad performance against tumor heterogeneity.

**TCGA-NSCLC**: This dataset has more positive tiles than CAMELYON16, accounting for above 80% per slide. Therefore, the main challenge is to distinguish the fine-grained patterns between subtypes. Here, we set $r_{1}$ and $r_{2}$ as 0.1 and 0.01, respectively. As shown in Table 1, our method surpasses the other methods by at least 1.57% on accuracy in terms of AUC, TransMIL achieves competitive performance, surpassing us only by 0.07%. Our method still outperforms the other methods by at least 1.4%. This demonstrates that our MDMIL has a powerful ability for fine-grained feature mining. Even for the subtypes with minor differences, MDMIL still can make more accurate diagnosis than other methods.

**TCGA-RCC**: This dataset, which is the same as the TCGA-NSCLC, has large areas of tumor region in the positive slide (average area > 80% per slide). It is a multi-classification problem with three subtypes. Apart from the challenge of detecting fine-grained patterns, another one is the data imbalance. KICH only has 70 training slides, much fewer than 337 slides of KIRC and 193 slides of KIRP. Here, we set $r_{1}$ and $r_{2}$ as 0.1 and 0.01, respectively. As shown in Table 1, our MDMIL can achieve superior performance to others even under such challenges. In terms of AUC, MDMIL achieves 99.44%, quite close to 100%. This significantly contributes to the memory-based contrastive loss, which consistently enforces distance constraints in the feature space. MDMIL also achieves the best accuracy, reaching 96.93%, much higher than any of the others. The outstanding performance of the proposed MDMIL method indicates that such methods may help bring steps closer to real-world applications.

We also compare the model size and Flops (floating point operations per second) of the algorithms in Supplementary Material Section S4. Considering both the Flops and performance, MDMIL stands out among these methods.

### 4.4 Ablation study

**Analysis of each component**: To verify the effectiveness of each proposed component, we present the ablation study of the internal query generation module (IQGM), deep projection layer (DPL), multiplex detection module (MDM), and memory-based contrastive loss ($L_{\text{CL}}$) on TCGA-NSCLC and TCGA-RCC datasets in Table 2. Here, we set β as 0 for a fair comparison. $\mathrm{Mode}l_{0}$ is the baseline method that utilizes a classification layer together with the *softmax* and max-pooling operations for the examination. $\mathrm{Mode}l_{1}$ adds the MDM on top of the transferred features. The same as DSMILLi et al. (2021), the classification and max-pooling operation help to generate the rough *IQ* for MDM. $\mathrm{Mode}l_{2}$ adds the DPL before MDM for feature transformation. Comparing $\mathrm{Mode}l_{0}$, $\mathrm{Mode}l_{1}$, and $\mathrm{Mode}l_{2}$, we can see significant improvements in both AUC and Accuracy. Especially on the TCGA-RCC dataset, the Accuracy improves from 76.09% to 94.12%, improving by 18%. From the comparison, we can conclude that our proposed MDM effectively finds critical instances that trigger the bag label and DPL is important for assisting the training. $\mathrm{Mode}l_{3}$ adds the IQGM in place of the max-pooling operation. It is obvious that with the assistance of IQGM, we can get a more reliable *IQ* for MDM and indirectly improve the instance mining ability of MDM. $\mathrm{Mode}l_{4}$ adds $L_{\text{CL}}$ on $\mathrm{Mode}l_{2}$. With the constraints in the feature space, the Accuracy and AUC improve on both TCGA-NSCLC and TCGA-RCC datasets. $\mathrm{Mode}l_{5}$ is the MDMIL that consists of IQGM, MDM, and $L_{\text{CL}}$. $\mathrm{Mode}l_{5}$ achieves the best performance, demonstrating that each module can work independently and corporately.

**Table 2:** Ablation study on the TCGA-NSCLC and TCGA-RCC datasets

					TCGA-NSCLC		TCGA-RCC	
	MDM	DPL	IQGM	$L_{\text{CL}}$	Accuracy	AUC	Accuracy	AUC
0	**✗**	**✗**	**✗**	**✗**	0.8038	0.8918	0.7609	0.9108
1	✔	**✗**	**✗**	**✗**	0.8341	0.9200	0.8930	0.9884
2	✔	✔	**✗**	**✗**	0.8768	0.9432	0.9412	0.9906
3	✔	✔	✔	**✗**	0.8957	0.9442	0.9305	0.9913
4	✔	✔	**✗**	✔	0.8815	0.9365	0.9519	0.9950
5	✔	✔	✔	✔	0.8957	0.9515	0.9679	0.9948

Specifically, we evaluate the performance of the proposed MDM, IQGM, and $L_{\text{CL}}$ in terms of accuracy and AUC.

**Analysis of** β **in IQGM**: β is a critical hyperparameter in IQGM. By acting as the bias, it can provide prior knowledge for the network. In Table 3, we present the experiment results with different β, ranging from −0.1 to 0.1. Specifically, we take the CAMELYON16 dataset as an example. As said before, the metastasis region only covers a small portion of WSIs; with the instance imbalance issue, the classification layer trends to give higher probabilities for normal patches and lower probabilities for metastasis ones. Therefore, by subtracting β (<0) to the *cf* of the metastasis subtype (rather than $cf_{\text{max}}$), we can give metastasis patches more attention and avoid critical features being ignored. As for the TCGA dataset, since tumor tissue covers much areas of WSIs, the classification layer in IQGM will not be troubled by the instance imbalance issue within WSIs. β is taken as a margin for enhancing the reliability of IQ. As shown in Table 3, when β is −0.1, we can achieve the best performance on CAMELYON16 datasets. However, when β>0, the performance drops dramatically, demonstrating that β is effective under the instance imbalance circumstances. As for the two TCGA datasets, when β>0, the performance becomes better and more stable, which proves it can improve the reliability of IQ.

**Table 3:** Analysis of β in MDM on the CAMELYON16, TCGA-NSCLC, and TCGA-RCC datasets in terms of accuracy and AUC for performance evaluation.

	CAMEYLON16		TCGA-NSCLC		TCGA-RCC	
β	Accuracy	AUC	Accuracy	AUC	Accuracy	AUC
−0.1	**0.9158**	**0.9669**	0.8830	0.9374	0.9519	**0.9951**
−0.05	0.9141	0.9664	0.8912	0.9429	0.9626	0.9950
0.0	0.8750	0.9357	0.8957	0.9515	0.9679	0.9948
0.05	0.8672	0.9445	**0.9052**	**0.9596**	**0.9693**	0.9944
0.1	0.8359	0.9057	0.8830	0.9374	**0.9693**	0.9944

### 4.5 Attention and feature distribution visualization

In Supplementary Material Section S3, we interpret the proposed algorithm by visualizing the attention regions on WSI and present the feature distribution before and after DPL. We conclude that our MDMIL can automatically detect critical regions in tissue without patch-level annotation and DPL is significant in deep transfer learning for relieving the feature distribution challenge.

## 5 Summary

In this study, we propose multiplex-detection-based multiple instance learning termed MDMIL to tackle the tumor heterogeneity issue in WSI classification. Specifically, MDMIL is constructed by the IQGM and the multiplex detection module (MDM) and assisted by the memory-based contrastive loss in the training phase. The proposed IQGM and MDM jointly detect critical instances through generated IQ and predefined VQ in the form of internal and external assistance. Since IQ and VQ can be complementary with each other, the multiplex detection strategy significantly improves the model’s perception ability towards critical instances. Meanwhile, the memory-based contrastive loss helps to reach consistency on various phenotypes in the feature space. Our novel network and loss function jointly achieve excellent robustness towards tumor heterogeneity. We conduct experiments on three computational pathology datasets. MDMIL’s outstanding performance verifies its ability against tumor heterogeneity. Meanwhile, our ablation study demonstrates each proposed module can work independently and cooperatively. The limitation of MDMIL lies in losing the contextual information during the computation. Therefore, for tasks like survival analysis, which need to analyze tumor micromovement and interactions between tumor cells and their neighbors, MDMIL may not perform as well as contextual-reserved methods.

## Supplementary Material

btad114_Supplementary_Data

## Data Availability

The datasets underlying this article are available in The Cancer Genome Atlas (TCGA) at [https://portal.gdc.cancer.gov/] and the CAMELYON16 challenge at [https://camelyon16.grand-challenge.org/].
