# Supplementary material for: Targeting tumor heterogeneity: multiplex-detection-based multiple instance learning for whole slide image classification
Source: Bioinformatics. 2023 Mar 2;39(3):btad114. doi: 10.1093/bioinformatics/btad114 (PMC10023223; doi:10.1093/bioinformatics/btad114)
Supplement: btad114_Supplementary_Data [file btad114_supplementary_data.zip › Supplementary_MDMIL.docx]

**Targeting Tumor Heterogeneity: Multiplex-Detection-Based Multiple Instance Learning for Whole Slide Image Classification**

Zhikang Wang^1^, Yue Bi^1^, Tong Pan^1^, Xiaoyu Wang^1^, Chris Bain^2^, Richard Bassed^3^, Seiya Imoto^4^, Jianhua Yao^5^, Roger J. Daly^1^, and Jiangning Song^1, *^

^1^Biomedicine Discovery Institute and Department of Biochemistry and Molecular Biology, Monash University, Melbourne, VIC, Australia; ^2^Faculty of Information Technology, Monash University, Melbourne, VIC, Australia; ^3^Victorian Institute of Forensic Medicine, Australia; ^4^Human Genome Center, Institute of Medical Science, University of Tokyo, Japan; ^5^Tencent AI Lab, Shenzhen, China.

*To whom correspondence should be addressed: [Jiangning.Song@monash.edu](mailto:Jiangning.Song@monash.edu)

**Supplementary materials**

1. Post-layer Normalization Feed-Forward Network (FFN)

**Supplementary Figure 1**. Overview of the feed-forward network (FFN) (a) and its FFN module (b).

This subsection gives details of Post-layer Normalization Feed-Forward Network (FFN) Xiong, R. *et al* (2022). As shown in Fig.1 (a), a residual connection is applied on the original and transformed features. As for the FFN module Fig.1 (b), we first adopt a linear layer to expand the feature dimension, and then feed the expanded features into GEGLU. GEGLU projects the features into two parts and adopts the activation function GELU to nonlinear the features. Then we adopt the multiplication of the two features as output. Functionally, GEGLU can be expressed by:

$$GEGLU\left( f_{ag} \right)=GELU\left( f_{ag}W+b \right)\cdot\left( f_{ag}V+c \right),$$

where $W,V$ are the transform matrixes, and $b,c$ are the biases. A linear layer is applied on the features for recalibration.

2. Implementation Details and Evaluation Metrics

Here, we train our network in an end-to-end fashion through the Adam optimizer with a weight decay of 5e-3. The learning rate is initialized as 2e-4 with cosine learning rate decay. In each iteration, only one bag of features corresponding to one WSI will be fed as input data. To increase the data diversity, we randomly discard 0~10% instances as an augmentation strategy in the training phase. For the two TCGA datasets, we only train the model for 100 epochs. As for the CAMELYON16 dataset, considering the fewer critical instances in each WSI, we train the model for 200 epochs. All the experiments are conducted on one NVIDIA GeForce RTX 3090 Graphic Card.

In this paper, we report the area under curve (AUC) scores and accuracy for evaluation. AUC is a graph showing the performance of a classification model at all classification thresholds. Accuracy gives the percentage of correct classifications.

3. Attention and Feature Distribution Visualization

**Supplementary Figure 2**. Attention visualization on both LUSC and LUAD WSIs. The first (a,d) and second (b,e) columns present the original WSIs segmented with green and blue curves, and images mapped with generated attention scores from MDCA, respectively. The last column (c,f) gives high score instances cropped by the yellow boxes on original WSIs.

Human readable interpretability of the trained deep neural networks can validate the model's predictive basis aligned with pathologists' knowledge. Besides, attention maps are also a functional tool to assist human-in-the-loop clinical diagnosis. In Fig.2, we visualize the attention scores and critical instances of WSIs in TCGA-NSCLC dataset. Here, we take two WSIs of LUSC and LUAD as examples. The first column (a, d) gives the original WSIs after the segmentation algorithm. The green curves circle the tissue region, and the blue ones eliminate the space inside. The second column (b,e) maps the attention scores generated by MDCA onto the images. Specifically, the attention scores are the combination of the attention matrixes ($mt_{1}$and $mt_{2}$) generated by *IQ* and *VQ*. Before mapping, we conduct the normalization operation on the attention matrix to scale the value to 0~1. In the third column (c,f), we present the high-score tissues from the yellow boxes in (a,b). From (b,e), we can see that, although pixel-level or patch-level annotation was never utilized to assist the training of the network, our proposed MDMIL still can highlight the critical instances correlated to the tumor subtype. Besides, by comparing (c,f), we can see two different varieties of unique patterns of LUSC and LUAD. This finding demonstrates that MDMIL has the potential to be used for meaningful WSI interpretability and visualization in cancer subtyping problems for clinical or research purposes.

**Supplementary Figure 3**. Feature distribution before and after DPL on CAMELYON16 (a,b) and TCGA-NSCLC (c,d) datasets. Here, we take T-SNE for visualization in the feature space. It is obvious that DPL helps to separate features of different subtypes apart, alleviating the learning pressure of subsequent modules.

In Fig. 3, we visualize the feature distribution before and after DPL through T-SNE Van der Maaten, L. *et al* (2008) on CAMELYON16 (a,b) and TCGA-NSCLC (c,d) datasets. Specifically, for each WSI, we randomly select 20 instances from the top 20% ones of the labelled subtype through the first classification layer. For the CAMELYON16 dataset Fig.3 (a), there is a massive difference in feature distribution on positive samples (metastasis) and negative samples (un-metastasis); a simple decision surface cannot distinguish them. With the application of DPL Fig.3 (b), although still with independent clusters (circled by green curves), the disorder situation alleviates significantly. As for the TCGA-NSCLC dataset Fig.3 (c), the features are more distinguishable due to the discrepancy in critical patterns of LUAD and LUSC. However, there remain fewer overlapping and many independent clusters (circled by green curves). Our DPL helps to tackle this issue and separate them in the feature space Fig.3 (d).

All in all, DPL is critical in the deep transfer learning architecture and can provide more discriminative features for the following modules.

4. Model Size and Calculation Evaluation

**Supplementary Table 1.** Model size and flops. The flops are measured with the number of instances of a bag being 10,000, and the instance feature extraction is not considered in this table.

| Methods | Model Size | Flops |
| --- | --- | --- |
| ABMIL | 927.6K | 7.86G |
| DSMIL | 855.7K | 1.15G |
| TransMIL | 2.67M | 27.55G |
| MDMIL | 6.38M | 10.56G |

In this subsection, we compare our proposed MDMIL with other state-of-the-art methods on model size and flops. As shown in Table.1, attention-based algorithms with few layers (ABMIL Ilse, M. *et al* (2018) and DSMIL Li, B. *et al* (2021)) have few parameters and smaller flops. However, this also results in bad generalization ability and performance. Compared with the transformer-based algorithm TransMIL Shao, Z. *et al*, although MDMIL has more parameters, since we reduce the dimension on features and instances through DPL and MDMC at the very start of the model, our flops are much smaller. Considering both the Flops and performance, MDMIL stands out among these methods.

**References**:

Xiong, R. *et al* (2022). "On layer normalization in the transformer architecture" International Conference on Machine Learning. PMLR, 2020.

Van der Maaten, L. *et al* (2008). "Visualizing data using t-SNE." Journal of machine learning research 9.11 (2008).

Ilse, M. *et al* (2018). "Attention-based deep multiple instance learning." International conference on machine learning. PMLR, 2018.

Li, B. *et al* (2021). "Dual-stream multiple instance learning network for whole slide image classification with self-supervised contrastive learning." Proceedings of the IEEE conference on computer vision and pattern recognition. 2021.

Shao, Z. *et al*. "Transmil: Transformer based correlated multiple instance learning for whole slide image classification." Advances in Neural Information Processing Systems 34 (2021): 2136-2147.
